# The Flaxseed-Derived Lignan Phenolic Secoisolariciresinol Diglucoside (SDG) Protects Non-Malignant Lung Cells from Radiation Damage

**DOI:** 10.3390/ijms17010007

**Published:** 2015-12-22

**Authors:** Anastasia Velalopoulou, Sonia Tyagi, Ralph A. Pietrofesa, Evguenia Arguiri, Melpo Christofidou-Solomidou

**Affiliations:** Department of Medicine, Pulmonary, Allergy and Critical Care Division, University of Pennsylvania, 3615 Civic Center Boulevard, Abramson Research Building, Suite 1016C, Philadelphia, PA 19104, USA; avela@mail.med.upenn.edu (A.V.); tyagisonia@gmail.com (S.T.); ralphp@mail.med.upenn.edu (R.A.P.); evguenia@mail.med.upenn.edu (E.A.)

**Keywords:** antioxidant, DNA damage, flaxseed, ionizing radiation, lignan, phenolic, radioprotection, SDG

## Abstract

Plant phenolic compounds are common dietary antioxidants that possess antioxidant and anti-inflammatory properties. Flaxseed (FS) has been reported to be radioprotective in murine models of oxidative lung damage. Flaxseed’s protective properties are attributed to its main biphenolic lignan, secoisolariciresinol diglucoside (SDG). SDG is a free radical scavenger, shown in cell free systems to protect DNA from radiation-induced damage. The objective of this study was to investigate the *in vitro* radioprotective efficacy of SDG in murine lung cells. Protection against irradiation (IR)-induced DNA double and single strand breaks was assessed by **γ-**H2AX labeling and alkaline comet assay, respectively. The role of SDG in modulating the levels of cytoprotective enzymes was evaluated by qPCR and confirmed by Western blotting. Additionally, effects of SDG on clonogenic survival of irradiated cells were evaluated. SDG protected cells from IR-induced death and ameliorated DNA damage by reducing mean comet tail length and percentage of **γ**-H2AX positive cells. Importantly, SDG significantly increased gene and protein levels of antioxidant HO-1, GSTM1 and NQO1. Our results identify the potent radioprotective properties of the synthetic biphenolic SDG, preventing DNA damage and enhancing the antioxidant capacity of normal lung cells; thus, rendering SDG a potential radioprotector against radiation exposure.

## 1. Introduction

Fruits, vegetables, cereals and beverages contain more than 8000 natural compounds that are characterized as polyphenols [1]. Depending on the number of their phenolic rings and other structural characteristics, polyphenols are classified as phenolic acids, flavonoids, stilbenes and lignans [1]. Consumption of polyphenols and the bioavailability of their secondary metabolites has been related to the protection against a variety of pathological conditions including carcinogenesis, cardiovascular diseases, diabetes, osteoporosis and neurodegenerative diseases [1].

The generation of reactive oxygen species (ROS) deregulates the endogenous antioxidant mechanisms in cells, leading to macromolecule damage, characterized in part by lipid peroxidation, DNA-protein crosslinks, base modifications, adduct formation and DNA single- and double-strand breaks [2,3]. These modifications initiate complex signal transduction pathways such as those involved in DNA repair, cell cycle arrest and induction of apoptosis [4].

Protection of normal tissue from radiation-induced damage such as that occurring by accidental exposure or as side effect of conventional radiotherapy to treat malignancies [5] is of great importance. Currently, there is an unmet need to develop a safe and effective radioprotecting pharmacological agent [6,7]. A large number of potential radioprotective agents have been reported, but their high cost, serious side effects and toxicity have limited their clinical usefulness [4,8]. On the other hand, natural compounds, especially polyphenolics, have been tested as potential radioprotectors, due to their antioxidant, anti-inflammatory, antimicrobial, immunomodulatory and anti-carcinogenic activities as well as their low toxicity profile and high availability. Polyphenolic compounds can act as free radical scavengers and inhibitors of lipid peroxidation. They upregulate pro-survival factors and cytoprotective antioxidant enzymes, as well as modulate DNA repair [6,9]. 

For the past few years, our group has been evaluating the protective effects of dietary flaxseed (FS) supplementation in preclinical murine models of oxidative lung damage such as hyperoxia, acid aspiration injury [10], and ischemia/reperfusion injury. We determined that the protective effects of FS may be due in part to its ability to enhance antioxidant enzyme expression in lung tissues [10,11]. Importantly, dietary FS ameliorated the adverse effects of thoracic radiation when given both prior to exposure [12] as well as post-exposure [13]. In these studies, dietary flaxseed decreased radiation-induced oxidative lung tissue damage, decreased lung inflammation and prevented pulmonary fibrosis.

We have further characterized the radioprotective effects of the lignan component (FLC) of wholegrain flaxseed, enriched in the phenolic secoisolariciresinol diglucoside (SDG). The antioxidant and free radical scavenging properties of SDG are well documented [14,15] which is of paramount importance as the free radical scavenging ability of a compound can be directly related to its radioprotective efficacy. In an early pilot study on lung endothelial cells, SDG exhibited free radical scavenging properties when cells were exposed to gamma-irradiation [12]. Importantly, the entire flaxseed lignan component (FLC) enriched in SDG, mediated radioprotection [16] and radiation mitigation [17] in mice. We recently chemically synthesized SDG (LGM2605) to allow scalable synthesis for evaluation in large scale experiments [18]. We confirmed potent free radical scavenging and antioxidant properties of LGM2605, comparable to the commercially available SDG. We extended our evaluation of synthetic SDG and further confirm DNA radioprotective properties of the synthetic phenolic in cell free systems [19]. However, characterization of the radioprotective properties of SDG in cells or tissues has not yet been evaluated. This study was performed in order to determine the radioprotective ability of the biphenolic SDG in three lung cell types (endothelial, epithelial and fibroblasts) against damaging gamma radiation. 

In this study, we show for the first time that SDG pre-treatment protects lung cells from radiation-induced DNA damage and increases their clonogenic survival. SDG also significantly boosts the endogenous antioxidant capacity of the lung cells, increasing the gene expression and protein levels of antioxidant enzymes, such as HO-1, GSTM1 and NQO1. Our findings identify the lignan biphenolic SDG as a potential radioprotective agent for normal lung cells.

## 2. Results

### 2.1. SDG Prevents Formation of DNA Single Strand Breaks in Irradiated Lung Cells

**Figure 1:**
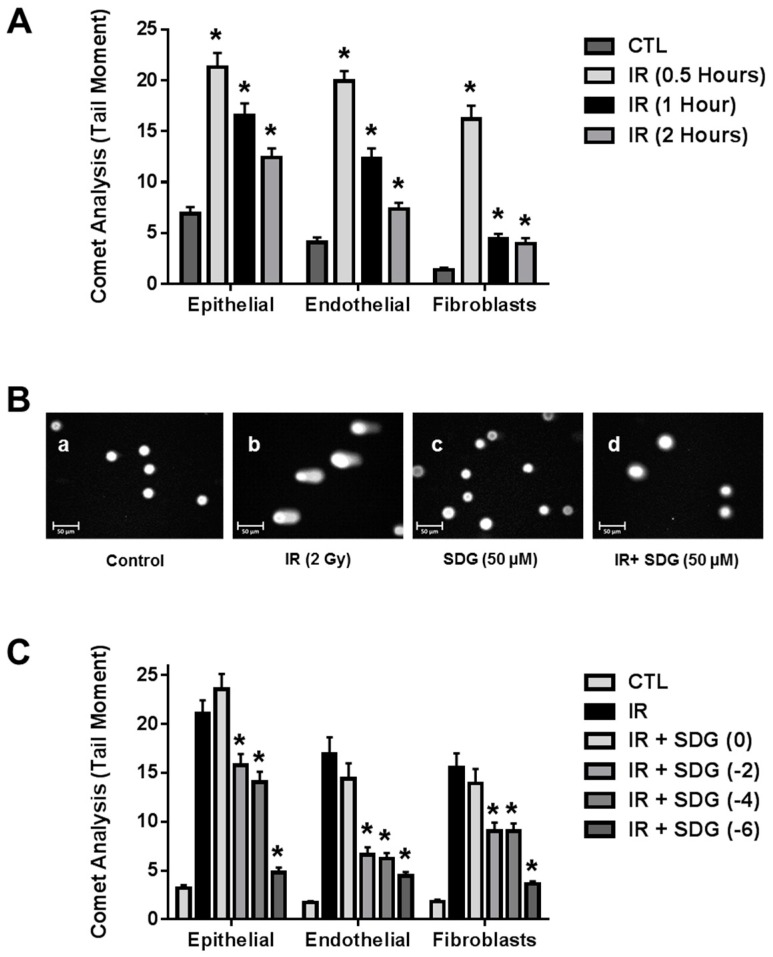
Evaluation of the radiation-induced DNA single-strand breaks (SSB) in lung cells using the alkaline comet assay. (**A**) Kinetic evaluation of DNA damage in irradiated lung cells (epithelial, endothelial and fibroblasts) with 2 Gy; * *p* ≤ 0.001 for non-irradiated controls *vs.* their respective irradiated cells; (**B**) Representative fluorescence photomicrograph of lung epithelial cells at 400× magnification. Cells were pre-treated with SDG (50 μM) and exposed to γ-rays (2 Gy). Image a: Control cells, Image b: SDG (50 μM), Image c: IR (2 Gy) after 30 min of exposure, and Image d: Cells pre-treated with SDG (50 μM, 6 h) and irradiated; (**C**) Effect of SDG (50 μM) treatment (0, 2, 4, and 6 h prior to irradiation) on irradiated lung cells. Data are represented as mean ± SEM. * indicates *p* < 0.05, for irradiated cells *vs.* SDG pre-treated irradiated cells.

We first performed a study to determine the kinetics of DNA damage in three cell types abundant in lung tissues (endothelial, epithelial, fibroblasts) following exposure to a radiobiologically relevant dose of 2 Gy. As expected, radiation exposure induced significant DNA damage, as evidenced by the increased tail moment, in all cell types compared to their respective non-irradiated control cells. DNA damage peaked at 30 min post irradiation. The extent of DNA damage decreased steadily thereafter. We therefore, selected the 30-min time-point for further studies evaluating SDG inhibition of radiation induced DNA damage (Figure 1A).

In a separate series of experiments, cells were pre-treated with SDG at various times prior to 2 Gy radiation exposure (0 h, 2 h, 4 h, and 6 h). Pre-treatment of cells with SDG (50 μM), at all-time intervals, significantly (*p* < 0.05) inhibited comet tail length in all cell types evaluated, an indication of decreased DNA damage. Maximum protection was observed with 6 h SDG pre-treatment (Figure 1C). We evaluated a longer pre-treatment time with SDG prior to radiation exposure (24 h), however, measurements of tail moment showed that there was no protection of epithelial cells (22.4 ± 1.64) and fibroblasts (12.6 ± 0.80), but a significant (*p* < 0.05) protection of endothelial cells (5.3 ± 0.55). A representative fluorescence photomicrograph depicting the formation of comet tails in irradiated epithelial cells with and without SDG treatment is shown in Figure 1B. Thus, the six-hour-period of pre-treatment was selected as optimal for all subsequent studies.

### 2.2. SDG Abrogates the Induction of γ-H2AX in Irradiated Murine Lung Cells

**Figure 2:**
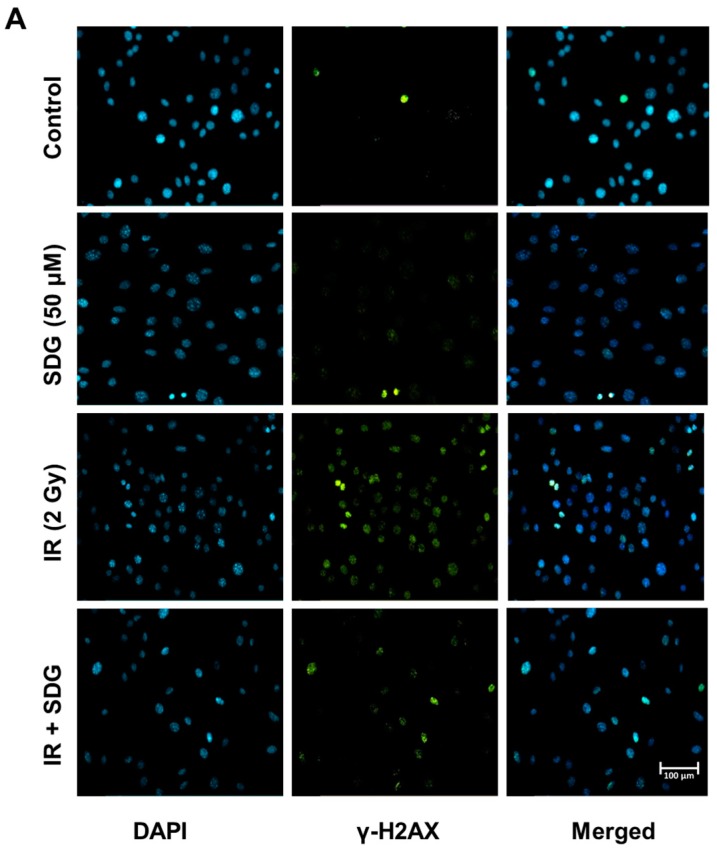

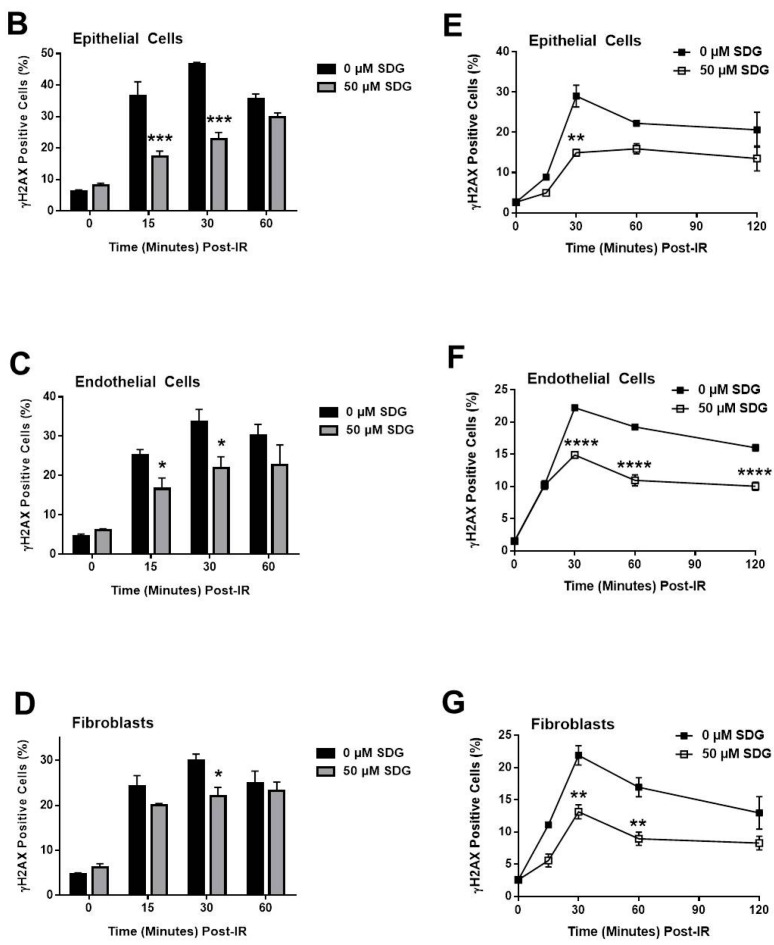
Evaluation of γ-Η2ΑΧ foci in irradiated murine epithelial cells, endothelial cells and wild-type fibroblasts. (**A**) Representative panels of immunofluorescence visualization of γ-H2AX foci (green) in murine lung epithelial cells at 200× magnification. Cells were pre-treated with SDG for 6 h and irradiated with γ-rays (2 Gy); (**B**–**D**) Evaluation of DNA damage by γ-Η2ΑΧ foci counting on (**B**) Epithelial Cells, (**C**) Endothelial Cells, and (**D**) Fibroblasts; (**E**–**G**) Flow cytometric (FACS) analysis of γ-H2AX foci in irradiated murine lung cells (**E**) Epithelial Cells, (**F**) Endothelial Cells, and (**G**) Fibroblasts. Cells were treated with SDG (50 μM) for 6 h and gamma-irradiated (2 Gy). At desired time interval, cells were processed for FACS analysis. Data were quantified using Summit software and is represented as mean ± SEM. * indicates *p* < 0.05, ** indicates *p* < 0.01, *** indicates *p* < 0.001, **** indicates *p* < 0.0001 for irradiated cells *vs.* SDG pre-treated irradiated cells.

Radiation-induced double-stranded breaks result in the phosphorylation of histone H2A variant H2AX [20] and is considered a reliable and sensitive marker of DNA damage. In order to evaluate the efficacy of SDG to protect lung cells from DNA damage, we assessed the formation of the γ-H2AX foci, after irradiation of SDG-pretreated lung cells, using standard microscopy-generated image analysis (Figure 2A–D) and further confirmed with flow cytometry (Figure 2E–G). Results of fluorescence microscopic analysis show that radiation (2 Gy) exposure led to a significant increase in the formation of γ-H2AX foci in all three cell types (Figure 2B–D). The number of foci/cell increased substantially by 15 min, peaked at 30 min post irradiation (46.7% ± 0.5%, 33.6% ± 3.2% and 30.0% ± 1.4% of γ-H2AX-positive cells, for epithelial, endothelial and fibroblasts, respectively) while numbers decreased notably within one hour of exposure albeit still significantly higher than non-irradiated control cells. All values were significantly higher (Figure 2B–D) compared to their respective non-irradiated control cells (*p* < 0.005 for all cell types). SDG pre-treatment (six hours prior IR based on findings from the above studies) significantly decreased the induction of γ-H2AX, as the number of γ-H2AX positive cells decreased to 22.7% ± 2.17%, 21.92% ± 2.88% and 22.1% ± 1.9% in irradiated epithelial cells, endothelial cells and fibroblasts, respectively, (*p* < 0.05 for epithelial and *p* < 0.05 for endothelial and fibroblasts). SDG pre-treatment equally protected all three types of lung cells from radiation-induced DNA strand breaks. Figure 2Α depicts a representative fluorescence photomicrograph of microscopic analysis of γ-H2AX positive cells in lung epithelial cells. The protective effect of SDG on blunting the induction of γ-H2AX positive cells after radiation exposure was further confirmed using flow cytometry. As expected, a similar pattern in the induction of γ-H2AX-positive cells was observed post-irradiation which was significantly abrogated by SDG (Figure 2E–G).

### 2.3. SDG Treatment Increases Colony Forming Ability of Irradiated Lung Cells

**Figure 3:**
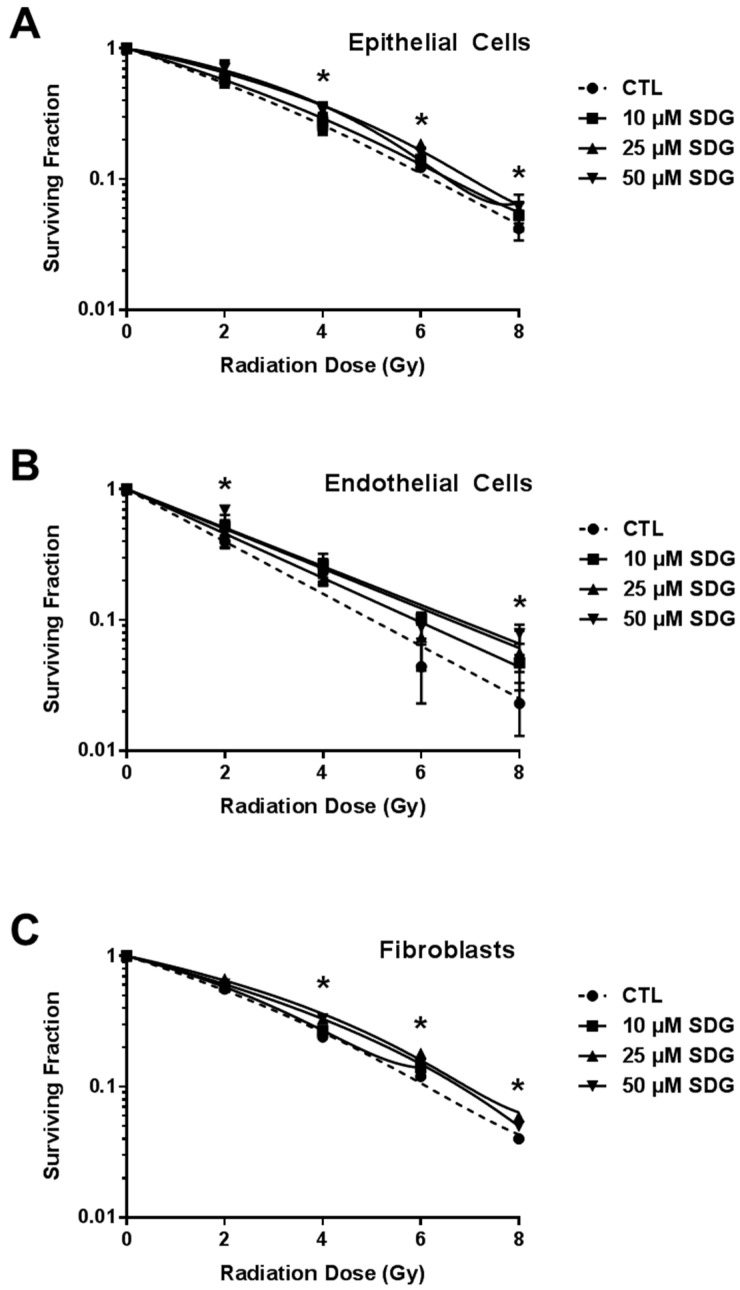
Effect of SDG treatment on radiation dose response of murine lung cells (**A**) Epithelial Cells; (**B**) Endothelial Cells; and (**C**) Fibroblasts. Cells were treated with different concentrations of SDG for six hours prior to gamma irradiation (0, 2, 4, 6, and 8 Gy). All visible colonies were counted two weeks post exposure and surviving fraction was normalized against non-irradiated control values. Data are represented as mean ± SEM. * indicates *p* < 0.05, for irradiated cells *vs.* SDG pre-treated irradiated cells.

The clonogenic survival assay has been used widely to determine cellular reproductive death after a cell undergoes any genotoxic stress following exposure to ionizing radiation. In this study, SDG (10–50 μM) protective activity over clonogenicity of lung cells (epithelial cells, endothelial cells and fibroblasts, respectively) was evaluated. The SDG doses were selected based on studies by Kitts *et al*. investigating the antioxidant and DNA-protective action of flaxseed lignans *in vitro* [21]. Results show that SDG (10–50 µM) alone did not elicit any adverse effect on the colony forming ability of all the three cell types as compared to their respective untreated control cells (100%) (Figure 3). Radiation treatment significantly (*p* ≤ 0.01) reduced the colony forming ability of epithelial and endothelial cells in a dose-dependent manner. When cells were treated with SDG prior to irradiation, the surviving fraction was enhanced significantly in all the treatment groups (Figure 3A,B). Maximum protection against radiation–induced loss of clonogenicity in fibroblasts was observed when cells were pre-treated with 50 μM SDG (Figure 3C).

### 2.4. SDG Treatment Modifies the Gene and Protein Levels of Antioxidant Enzymes in Murine Lung Cells

**Figure 4:**
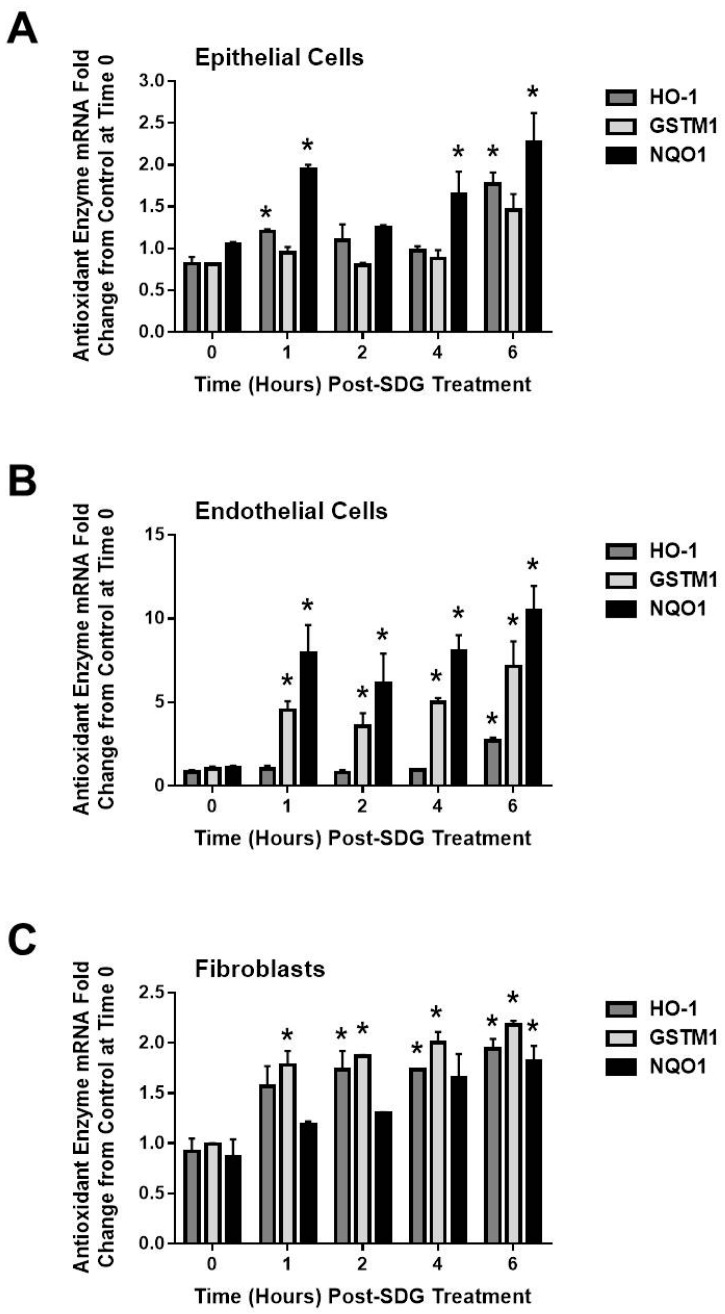
Evaluation of the effect of SDG treatment on genes encoding the antioxidant/cytoprotective enzymes, HO-1, GSTM1 and NQO1. (**A**) Epithelial Cells; (**B**) Endothelial Cells and (**C**) Fibroblasts. Cells after being treated with SDG (50 µM), were evaluated kinetically at 1, 2, 4, and 6 h post SDG treatment. Total RNA was isolated at desired time points and evaluated by qPCR analysis for *HO-1*, *GSTM1* and *NQO1* gene expression. Analysis was performed in triplicate and gene expression was normalized to 18S ribosomal RNA. * indicates *p* < 0.05, for irradiated cells *vs.* SDG pre-treated irradiated cells.

Our aim was to elucidate whether treatment of cells with SDG prior to irradiation upregulates the gene levels of representative antioxidant/cytoprotective enzymes [22] and determine the kinetics of gene expression in response to SDG, using qPCR. We incubated the three cell types (epithelial, endothelial and fibroblasts) with SDG (50 μΜ) and we evaluated the mRNA levels of HO-1, GSTM1 and NQO1, at 1, 2, 4 and 6 h post SDG treatment, in all three cell types (epithelial, endothelial and fibroblasts (Figure 4). We observed that SDG increases the mRNA levels of all three antioxidants enzymes, over the period of 6 hours, in all cell types tested. Interestingly, endothelial cells presented much higher change in their antioxidant enzyme mRNA levels post SDG treatment compared to the other cell types.

**Figure 5:**
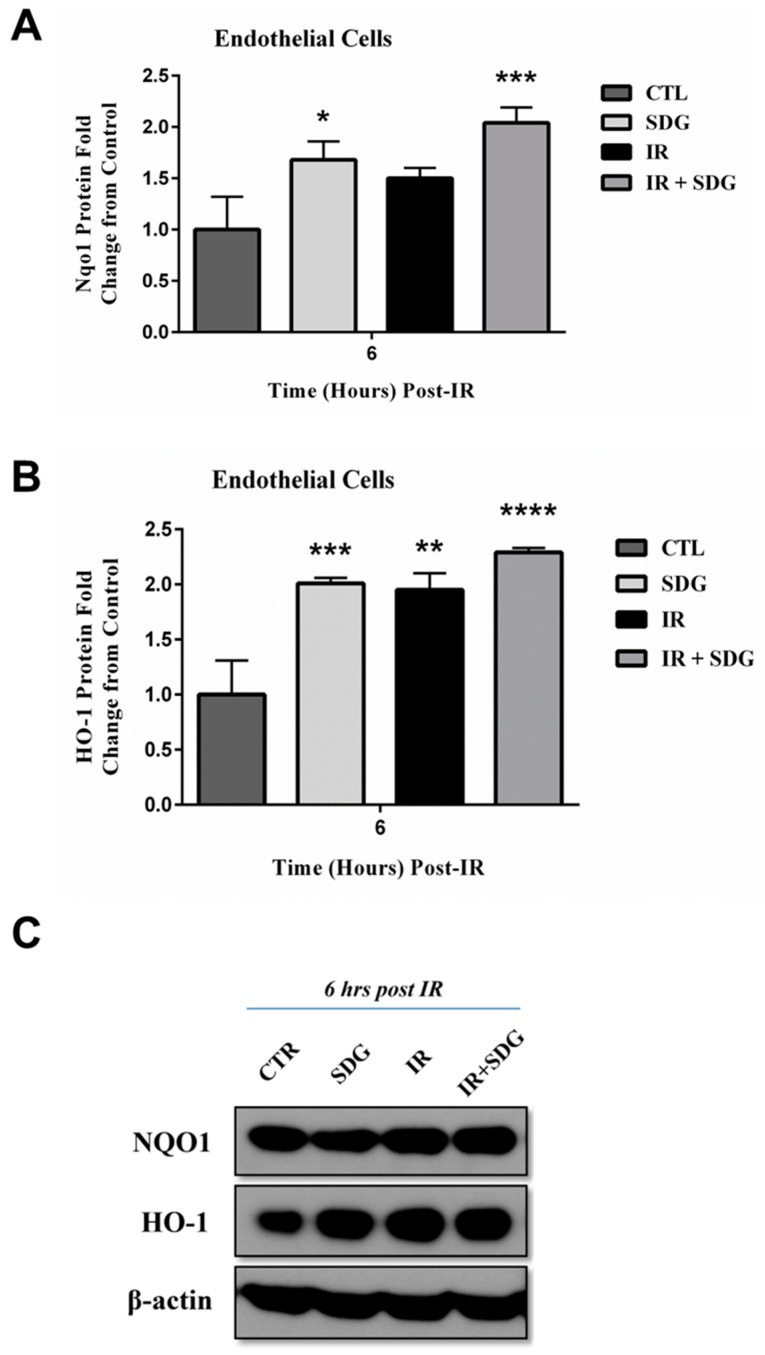
Western blot analysis of SDG-induced changes in levels of antioxidant/cytoprotective enzymes HO-1 and NQO1, in endothelial cells, after radiation (2 Gy). Murine lung cells (epithelial cells) were treated with SDG (50 µM) for six hours prior to exposure to γ-rays. Cells were harvested at six hours post-radiation. (**A**) Densitometric analysis of Nqo1 with normalization to β-actin; (**B**) Densitometric analysis of HO-1 with normalization to β-actin; (**C**) Representative Western blot images. Experiment was repeated three times and data are represented as mean fold change ± SEM. * *p* < 0.05, ** *p* < 0.01, *** *p* < 0.001 and **** *p* < 0.0001 for all groups *vs.* untreated cells.

We therefore chose this cell type, to further investigate whether pre-treatment of endothelial cells with SDG (50 μM) for 6 h can lead in an increased antioxidant protein expression, at 6 h post irradiation of cells with γ-rays (2 Gy). Overall, protein levels of both NQO1 and HO-1 were significantly increased (2- and 2.3-fold, respectively) in SDG-pretreated cells at 6 h post radiation (Figure 5A,B depict band density quantification whereas Figure 5C depicts representative blots).

## 3. Discussion

**Figure 6:**
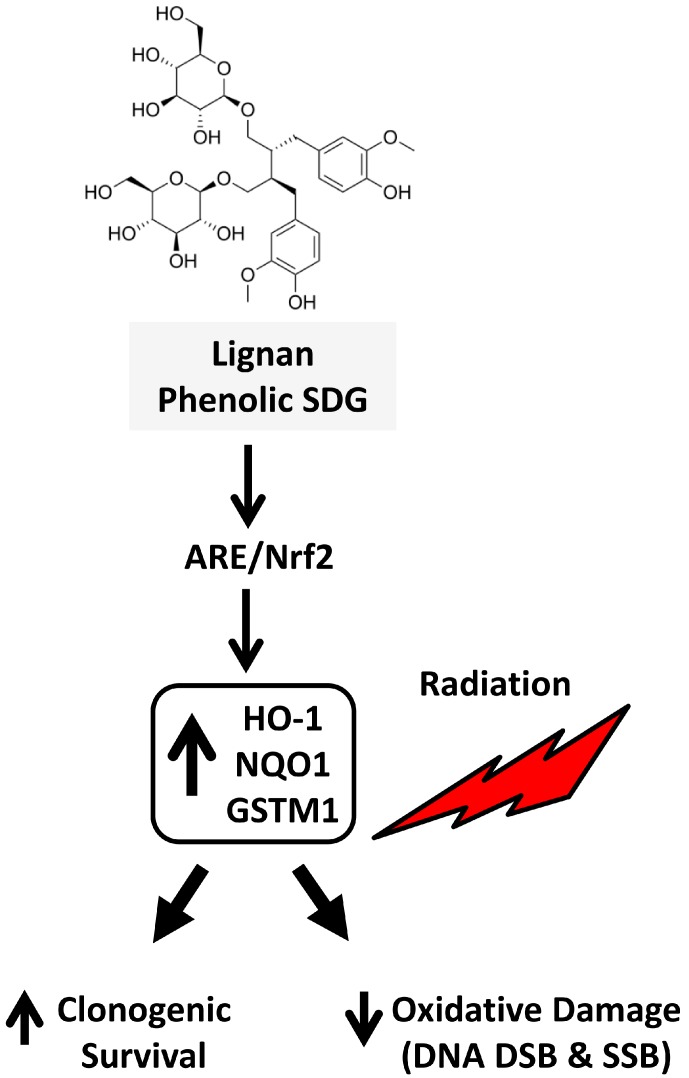
Schematic representation of possible mechanism by which lignan phenolic SDG (LGM2605) protects lung cells against radiation exposure. SDG upregulates the phase II cytoprotective enzymes, HO-1, NQO1 and the gene levels of GSTM1; thus, when the non-malignant lung cells are exposed to ionizing radiation, less oxidative damage is induced, leading to increased clonogenic survival of the irradiated cells.

In the current study, we demonstrated for the first time that the lignan phenolic SDG can protect murine lung cells against radiation-induced oxidative damage. We observed that pre-treatment of cells with SDG decreased radiation-induced DNA strand breaks (single- and double-stranded breaks) and improved the overall cell survival as measured by clonogenic survival assay. Importantly, expression of the Nrf2-regulated enzymes, NQO1 and HO-1, was also altered by SDG treatment, indicating its important role in adaptation to oxidative stress (Figure 6).

Our results are concordant with our previous studies in cell-free systems, in which plasmid and calf thymus DNA were exposed to gamma radiation resulting in DNA fragments of low-molecular weight, which were prevented in a dose-dependence manner by SDG [19].

Polyphenols have the ability to protect normal tissue or cells from damaging effects of radiation by reducing ROS mediated oxidative DNA damage [23]. In the present study, we evaluated the role of SDG against IR-induced DNA strand breaks in murine lung cells. While SSBs can more easily be repaired by the cell as compared to the DSBs, the latter are more likely to result in mutagenesis [24]. We report here that SDG protected cellular DNA from IR-induced SSBs and importantly, from DSBs in all three cell lines that tested. Our results are concordant with other reports that show a similar protective effect from radiation-induced both DNA SSB and DDB, for other phenolics such as chrysin [25] and epicatechin [26], resveratrol [27] and green tea catechin [28].

Α key biological response to oxidative stress is the activation of the Keap1/Nrf2/ARE pathway, which regulates many antioxidant and cytoprotective genes responsible for the cellular homeostasis [19]. Activation of this pathway prior to exposure to a genotoxic agent, such as ionizing radiation can be crucial for the fate of the cell. Here, pre-treatment of cells with the lignan phenolic SDG upregulated Keap1/Nrf2/ARE-regulated protective enzymes. Our group has previously shown that an SDG-rich diet (FLC) upregulates the antioxidant enzymes, HO-1 and NQO1, in a murine lung model of radiation. Findings of the current study support the dual role of SDG in free-radical scavenging and antioxidant defense boost [12,14], in protecting from radiation-induced DNA damage in normal cells.

SDG, similarly to other phenolic compounds, such as apigenin [29], hesperidin [30], silibinin [31] and epigallocatechin gallate (EGCG) [28], showed radioprotection by scavenging harmful free radicals or by increasing the cell endogenous antioxidant defense, such as trans-resveratrol [27], EGCG [28] and euticoside C [32]. However, despite over 50 years of research, most agents have not proceeded past pre-clinical evaluation or have not been evaluated in large trials [33]. Numerous natural products and their isolated purified bioactive ingredients have been shown to have antioxidant and anti-inflammatory potential [34,35], properties useful for a variety of pathological conditions, but most are not tolerated in humans at the concentrations required to achieve clinical efficacy [36]. In contrast, SDG is well tolerated in humans, its clinical efficacy was demonstrated in a wide range of pathological conditions with oxidative and inflammatory components. SDG formulations (FLC) were shown in preclinical models to be radioprotective in lung with antioxidant, anti-inflammatory and antifibrotic activity, while not inhibiting tumor killing by radiation [16]. Importantly, SDG formulations were safely administered to humans for 6–24 weeks with no reported toxicity and secondary lignan metabolites were detectable in biological fluids [37,38]. We have proceeded to chemically synthesize SDG (LGM2605) to enable preclinical evaluation in animal models of radiotherapy as well as subsequently in Phase I clinical trials [18,19].

The lignan phenolic SDG can be considered as a potential protective agent against radiation-induced oxidative damage to non-malignant lung cells. The protective effects of SDG from radiation damage in cells are mediated in part by free-radical scavenging and the boost of antioxidant defenses. Further studies are needed to validate SDG as an effective radioprotective agent with clinical usefulness. 

## 4. Materials and Methods

### 4.1. Cell Lines

Fibroblasts and endothelial cells were isolated from C57/Bl6 mouse as described previously [39]. For fibroblast isolation, mouse lungs were harvested, minced, and incubated with dispase (2 mg/mL) for 45 min. Cell suspension was plated out and fibroblasts were cultured as described previously. Cells were used while in passages 3–10. Pulmonary microvascular endothelial cells (PMVEC) were isolated from murine lungs as described previously [40]. Briefly, freshly harvested mouse lungs were treated with collagenase followed by isolation of cells by adherence to magnetic beads coated with mAb to platelet endothelial cell adhesion molecule (PECAM). Epithelial cells (C10) cells were originally derived from a normal BALB/c mouse lung explant and are non-tumorigenic [41], contact-inhibited, and have alveolar type 2 cell features at early passage (kindly provided by Dr. Alvin Malkinson, University of Colorado, Denver, CO, USA).

### 4.2. Reagents

Secoisolariciresinol Diglucoside (SDG) is commercially available (ChromaDex, Inc., Irvine, CA, USA) or was chemically synthesized based on a published synthetic pathway [18] and is designated as LGM2605. Initial experiments were performed with the commercially available SDG, which is costly and prohibitively expensive to permit animal dosing in long-term studies. Additionally, extraction methods to obtain large amounts of SDG are complex [42,43,44], require special resources and expertise and result in variable yields. A synthetic path to obtain SDG was thus pursued [18] to achieve the needed quality control, lot-to-lot uniformity, and dose and cost parameters consistent with pharmaceutical development. The chemically synthesized LGM2605 has remarkably similar antioxidant properties as the parent, natural compound [19]. Please note that since we have shown in several studies that commercially available SDG and synthetic SDG (LGM2605) behave identically with respect to their antioxidant and DNA radioprotective properties, we use SDG and LGM2605 interchangeably in the text. 

Comet assay kit was purchased from Trevigen, Inc., (Gaithersburg, MD, USA). P-Histone H2AX (rabbit mAb) was purchased from Cell Signaling Technology, Inc. (Danvers, MA, USA). Phosphate buffered saline (PBS), Bovine serum albumin (BSA), Dulbecco’s modified Eagle’s medium (DMEM) with l-glutamine, glucose 1 g/L, without sodium bicarbonate, HEPES buffer, trypsin, bovine serum albumin (BSA), ethylenediamine tetra acetic acid (EDTA), 4,6 diamidino 2-phenyl indole (DAPI), Fetal bovine serum (FBS), Collagenase, Triton-X 100 and dispase were purchased from Sigma-Aldrich, St. Louis, MO, USA.

### 4.3. COMET Analysis

Exponentially growing cells were cultured and treated with SDG (50 μM) at different time intervals prior to irradiation (2 Gy). Cells were processed for comet assay as per manufacturer’s instructions (Trevigen, Gaithersburg, MD, USA). Briefly, cells (1 × 10^5^ cells/mL in PBS) were mixed with LMAgarose^®^ (1:10, *v*/*v*) and immediately pipetted onto CometSlide™. Cells were then lysed (4 °C, 30 min) and kept in dark for unwinding (RT). Electrophoresis was done in a horizontal electrophoresis unit at 18 volts (200 Amp) for 25 min. Slides were washed twice with distilled water, fixed in 70% ethanol and dried at 45 °C. DNA was stained by SYBR green (Trevigen). At least 150 cells were scored per group. Visual analysis of cells and comet tail length was measured using Comet Image Analysis software (Comet Assay IV, Perceptive Instruments Ltd., Haverhill, MA, USA). Images were captured on an Olympus IX51 fluorescence microscope using a monochrome CCD FireWire camera with a 40× objective lens.

### 4.4. Immunostaining

For immunostaining of γ-H2AX, cells were plated on glass coverslips (5000 cells/coverslip), pre-treated (6 h) with 50 μM SDG and irradiated (2 Gy). At desired time interval, cells were fixed (4% para-formaldehyde), washed and blocked with PBST (PBS + 0.1% TritonX-100 containing 5% goat serum, 1% BSA). Cells were incubated with γ-H2AX antibody (1:200) overnight at 4 °C followed by washing with PBST (3 × 5 min) and incubation with secondary antibody (Alexa fluor^®^ 488, Invitrogen, CA, USA) for 1 h at RT. Nuclei were counterstained with DAPI and visualized under fluorescence microscope with a 20× objective lens.. Total cells (blue) γ-H2AX-positive cells (green) were counted per field and percentage of γ-H2AX positive cells were calculated. A minimum of 500 cells was counted for each treatment and the experiment was repeated twice.

### 4.5. Flow Cytometry for γ-H2AX

For FACS analysis, cells were trypsinized and washed with PBS. Cells were then fixed (Fix/Perm buffer, eBioscience, San Diego, CA, USA), for 45 min and washed thereafter using permeabilization wash buffer (BioLegend, San Diago, CA, USA). Cells were resuspended in 200 μL rabbit monoclonal phospho-histone γ-H2AX (Ser139) antibody conjugated to Alexa fluor^®^ 488 (1:100 *v*/*v*, Cell Signaling Technology, Danvers, MA, USA) and incubated for 30 min at 4 °C. Cells were washed again with wash buffer and analyzed. The CyAn ADP (Advanced Digital Processing) flow cytometer (Dako, Glostrup, Denmark) was used to measure γ-Η2AX and positive cells were quantified using Summit Software (Dako, Glostrup, Denmark).

### 4.6. Clonogenic Survival

Exponentially growing cells were plated as single cells and incubated overnight. Cells were treated with various doses of the lignan SDG (10–50 μM) 6 h prior to irradiation (2, 4, 6 and 8 Gy). Lignan dose was selected based on animal studies to be within the physiological levels reached in the blood circulation when 10% Flaxseed is ingested [10,13]. Cells were irradiated with a Mark 1 cesium (Cs-137) irradiator (J.L. Shepherd, San Fernando, CA, USA) at a dose rate of 1.7 Gy/min. Colonies were stained and counted 10 to 15 days after irradiation and surviving fraction was calculated.

### 4.7. Quantitative Real Time PCR (qPCR)

Quantitative polymerase chain reaction (qPCR) was performed as previously described [22]. Briefly, TaqMan^®^ Probe-Based Gene Expression Assays supplied by Applied Biosystems, Life Technologies (Carlsbad, CA, USA) were used. To evaluate the effect of SDG treatment on the mRNA expression of antioxidant genes, individual TaqMan^®^ gene expression assays were selected for antioxidant enzymes (heme oxygenase-1 (HO-1), NAD(P)H dehydrogenase, quinone 1 (NQO1), and glutathione *S*-transferase mu 1 (GSTM1)).

Briefly, cells were pre-treated with SDG (50 μM, 6 h) and irradiated (2 Gy). Total RNA was isolated from using RNeasy Plus Mini Kit (Qiagen, Valencia, CA, USA) and quantified using a NanoDrop 2000 (ThermoFisher Scientific, Waltham, MA, USA). Reverse transcription of RNA to cDNA was then performed on a Veriti^®^ Thermal Cycler using the High Capacity RNA to cDNA kit supplied by Applied Biosystems, Life Technologies (Carlsbad, CA, USA). qPCR was performed using 25 ng of cDNA per reaction well on a StepOnePlus™ Real-Time PCR System (Applied Biosystems, Life Technologies, Carlsbad, CA, USA). Gene expression data was normalized to 18S ribosomal RNA and calibrated to untreated control samples according to the ΔΔC_T_ method as shown previously [22].

### 4.8. Western Blotting

Cells were lysed in RIPA buffer containing protease inhibitor cocktail (Complete™, Mini, EDTA-free, Sigma-Aldrich). Total protein content was determined by *BCA* Protein Assay (Thermo Scientific). Total protein content was determined by *BCA* Protein Assay (Thermo Scientific). Samples were loaded on 8%–12% NuPAGE gel (Invitrogen, Carlsbad, CA, USA). Electrophoresis was performed at 200 V for 1 h. Transfer of proteins to PolyScreen PV transfer membrane (PerkinElmer Life Sciences, Boston, MA, USA) was performed for 2 h, at 25 volts. Membrane was blocked overnight in 5% non-fat dry milk in phosphate buffered saline. Protein levels of heme oxygenase 1 (HO-1) and NQO1 were detected using rabbit monoclonal anti-mouse antibodies, following manufacturer recommended dilutions (Abcam, Cambridge, MA, USA). Peroxidase-conjugated Donkey anti-rabbit IgG was used as a secondary antibody. (Jackson Laboratories, West Grove, PA, USA). Membranes were developed using Western Lighting Chemiluminescence Reagent Plus (PerkinElmer Life Sciences, Boston, MA, USA) and bands were visualized on standard X-ray film (HyBlot CL, Denville Scientific Inc., Metuchen, NJ, USA). Densitometric analysis of the bands was performed using ImageJ (NIH) software.

### 4.9. Statistics

Results are expressed as mean ± SEM. Survival curve for clonogenic assay was prepared using GraphPad Prism 6 software. Statistical differences among groups were determined using one-way analysis of variance (ANOVA). When statistically significant differences were found (*p* < 0.05), individual comparisons were made using the Bonferroni/Dunn test (Statview 4.0).

## Abbreviations

The following abbreviations are used in this manuscript:

DSBs: Double strand breaks
SSBs: Single strand breaks
FS: flaxseed
GSTM1: glutathione S-transferase mu 1
HO-1: heme oxygenase-1
IR: irradiation
NQO-1: NAD(P)H dehydrogenase, quinone 1
qPCR: quantitative real time PCR
ROS: reactive oxygen species
SDG: secoisolariciresinol diglucoside
SEM: standard error of the mean
